# From Volume to Value: A Systematic Review of Payment and Financial Incentive Models for Preventive Care Delivery in Primary Health Care

**DOI:** 10.7759/cureus.114915

**Published:** 2026-08-21

**Authors:** Anwar Alrashed, Dhari K Alobaidli, Waqas Alauddin

**Affiliations:** 1 Family Medicine, Dhaman Health Assurance Hospitals, Kuwait City, KWT; 2 Finance, Dhaman Health Assurance Hospitals, Kuwait City, KWT; 3 Physiology, Venkateswara Institute of Medical Sciences, Amroha , IND

**Keywords:** financial incentives, pay-for-performance, physician behaviour, physician payment models, preventive care, primary health care, value-based healthcare

## Abstract

Payment and financial incentive models in primary healthcare have undergone substantial transformation over the past two decades, shifting from volume-based reimbursement towards value-oriented approaches designed to improve healthcare quality and strengthen preventive care delivery. Despite their widespread implementation, evidence regarding their effectiveness remains inconsistent. This systematic review aimed to evaluate the effects of payment and financial incentive models on healthcare provider behavior, preventive care delivery, and related quality-of-care outcomes in primary healthcare settings.

A systematic search of electronic databases and grey literature was conducted in accordance with the Preferred Reporting Items for Systematic Reviews and Meta-Analyses (PRISMA) 2020 guidelines to identify randomised controlled trials (RCTs) and cluster-RCTs evaluating payment and financial incentive interventions. Study selection, data extraction, methodological quality assessment using the Cochrane Risk of Bias 2 (RoB 2) tool, and certainty of evidence assessment using the Grading of Recommendations Assessment, Development and Evaluation (GRADE) framework were performed using predefined criteria. Ten studies met the eligibility criteria and evaluated a range of payment and financial incentive models, including pay-for-performance, performance-based financing, direct facility financing, physician financial incentives, and patient-directed financial incentives.

Overall, the available evidence suggests that selected payment and financial incentive interventions may improve provider adherence to evidence-based clinical practice and strengthen preventive and process-of-care measures, including preventive screening, chronic disease management, vaccination uptake, and other quality-of-care indicators. However, improvements in patient-centered clinical outcomes were less consistent across studies. The methodological quality of the included trials was variable, with all studies demonstrating either some concerns or a high risk of bias, while the certainty of evidence ranged from very low to moderate. Payment and financial incentive interventions may strengthen selected aspects of preventive care delivery and quality of care in primary healthcare, although their effectiveness appears to depend on the design, target, and implementation of the intervention. The heterogeneity of intervention types, settings, and outcomes, together with methodological limitations among the included studies, limits the certainty and generalisability of the findings. Further high-quality multicenter RCTs with longer follow-up and standardized outcome measures are needed to determine the long-term effectiveness, cost-effectiveness, and generalisability of different payment and financial incentive models across diverse healthcare systems.

## Introduction and background

Primary healthcare forms the backbone of effective healthcare systems by delivering accessible, comprehensive, and continuous care throughout an individual's life. Beyond the diagnosis and treatment of illness, primary healthcare has a fundamental role in health promotion, disease prevention, early detection of illness, and long-term management of chronic diseases [1,2]. Countries with well-developed primary care systems generally achieve better health outcomes, greater equity in healthcare access, lower rates of preventable hospitalisation, and more efficient utilisation of healthcare resources than those that rely predominantly on specialist-based care [1,2]. In response to the growing burden of non-communicable diseases, population ageing, and escalating healthcare costs, improving the quality and effectiveness of preventive care has become an international health policy priority [1].

Among the many factors influencing the quality of primary care, the design of healthcare payment and financial incentive systems is particularly important because it can influence healthcare delivery, provider behaviour, resource utilisation, and clinical decision-making [3]. Payment and financing arrangements determine how healthcare providers, practices, facilities, and, in some programmes, patients are financially rewarded or supported within the healthcare system. These mechanisms may therefore influence patterns of care, adherence to recommended clinical practices, preventive service delivery, and utilisation of healthcare resources [3]. The traditional fee-for-service (FFS) model reimburses healthcare providers according to the number of services delivered. Although this approach may encourage service availability, it has been criticised for rewarding healthcare volume rather than healthcare value, potentially contributing to unnecessary investigations, fragmented care, and increased healthcare expenditure [3].

To address these challenges, healthcare systems have increasingly adopted alternative payment and financial incentive models designed to promote quality improvement and preventive healthcare [3,4]. These approaches include salary-based remuneration, capitation, bundled payment systems, shared savings programmes, pay-for-performance (P4P), performance-based financing, provider-level financial incentives, facility-level financing, and patient-directed financial incentives [3,4]. Unlike conventional volume-based reimbursement, many of these approaches seek to align financial incentives with predefined quality indicators, preventive care targets, service utilisation, or patient outcomes, thereby encouraging the delivery of higher-value healthcare [3,4]. The global movement towards value-based healthcare has further accelerated the implementation of such payment and financing reforms across both high-income and resource-constrained health systems.

Despite widespread implementation, the effectiveness of payment and financial incentive interventions remains uncertain. Financial incentives have been associated with improvements in preventive screening, chronic disease monitoring, vaccination uptake, and adherence to evidence-based clinical guidelines in some settings [4-6]. However, other studies have reported limited or inconsistent effects on patient-centred outcomes. Meanwhile, concerns have been raised regarding potential unintended consequences, including increased administrative burden, selective attention to incentivised activities, and reduced emphasis on aspects of care that are not financially rewarded [4-6]. These inconsistent findings suggest that the effects of payment and financial incentive interventions depend not only on the availability of financial incentives but also on their design, target, implementation, healthcare context, and interaction with organisational and patient-related factors.

Although several systematic reviews have examined physician remuneration and pay-for-performance programmes, many have synthesised evidence from observational studies alongside experimental research, making it difficult to distinguish causal effects from associations [3-5]. Furthermore, the evidence base has expanded considerably over the past decade with the publication of additional randomised controlled trials (RCTs) and cluster RCTs evaluating financial incentive programmes involving healthcare providers, primary care facilities, and patients. An updated synthesis focused exclusively on randomised evidence is therefore warranted to provide a more robust assessment of the effects of payment and financial incentive interventions on healthcare delivery and preventive practice.

Accordingly, this systematic review aimed to synthesise evidence from RCTs and cluster RCTs evaluating the effects of payment and financial incentive models on healthcare provider behaviour, preventive care delivery, and related quality-of-care outcomes in primary healthcare settings. In addition to summarising the effectiveness of different payment and incentive approaches, the review critically assessed the methodological quality of the included studies using the Cochrane Risk of Bias 2 (RoB 2) tool [7] and evaluated the certainty of evidence using the Grading of Recommendations Assessment, Development and Evaluation (GRADE) framework [8]. By focusing on randomised evidence, this review seeks to provide an updated understanding of how payment and financial incentive interventions influence preventive healthcare delivery and to generate evidence that may support future health financing policies, value-based primary care initiatives, and clinical decision-making.

## Review

Methods

Study Design and Protocol Registration

This systematic review was conducted in accordance with the Preferred Reporting Items for Systematic Reviews and Meta-Analyses (PRISMA) 2020 guidelines [9]. The review protocol was prospectively registered with the International Prospective Register of Systematic Reviews (PROSPERO) under registration number CRD420261461834. The registered protocol was used to guide eligibility criteria, study selection, data extraction, risk-of-bias assessment, and evidence synthesis.

Literature Search

A comprehensive literature search was conducted in PubMed/MEDLINE, Embase, Scopus, Web of Science, Cochrane CENTRAL, Cumulative Index to Nursing and Allied Health Literature (CINAHL), EBSCOhost, Google Scholar, and EconLit for studies published from January 2000 through December 2025. Grey literature and ongoing or registered studies were searched through ClinicalTrials.gov, the WHO International Clinical Trials Registry Platform (ICTRP), OpenGrey, PROSPERO, the Open Science Framework (OSF) Registries, the International Standard Randomised Controlled Trial Number (ISRCTN) Registry, and relevant organisational websites. The search strategy combined controlled vocabulary, including Medical Subject Headings (MeSH) and database-specific subject headings, with free-text terms related to payment and reimbursement models, financial incentives, provider or physician behaviour, primary healthcare, preventive care, and quality of care, using Boolean operators ("AND" and "OR"). Reference lists of included studies and relevant systematic reviews were also manually screened to identify potentially eligible studies [10-12]. The complete database-specific search strategies, search limits, and search dates are provided in Appendix A.

Eligibility Criteria and PICOS Framework

Eligibility criteria were predefined according to the Population, Intervention, Comparator, Outcomes, and Study Design (PICOS) framework [13]. For population: studies involving physicians, primary healthcare providers, primary care practices or facilities, and/or patients receiving or participating in primary healthcare services were eligible. Eligible interventions comprised payment and financial incentive models intended to influence healthcare delivery, provider behaviour, preventive care utilisation, or quality of care. These included FFS, capitation, salary-based remuneration, pay-for-performance, physician- or practice-level financial incentives, performance-based financing, facility-level financing, patient-directed financial incentives, and other value-based reimbursement or incentive strategies. Eligible comparator groups included usual care, routine reimbursement or financing arrangements, no additional financial incentive, or alternative payment and financial incentive models. Primary outcomes included provider or physician behaviour, adherence to evidence-based clinical guidelines, preventive healthcare delivery, preventive service utilisation, healthcare utilisation, patient engagement, and quality-of-care indicators. Patient-centred clinical outcomes were also considered where reported. The study design included only RCTs and cluster RCTs published in English. Observational studies, qualitative studies, systematic or narrative reviews, conference abstracts without sufficient outcome data, editorials, commentaries, protocols without available results, and non-English publications were excluded.

Study Selection

All records identified through the literature searches were imported into reference management software, and duplicate records were removed before screening. Two reviewers independently screened titles and abstracts against the predefined eligibility criteria, followed by independent full-text assessment of potentially eligible reports. Reasons for exclusion were documented at the full-text stage. Disagreements between reviewers were resolved through discussion, and a third reviewer was consulted when consensus could not be reached.

Data Extraction

Two reviewers independently extracted data using a standardised data extraction form. Extracted information included author and publication year, country, study design, healthcare setting, population characteristics, sample size, intervention type and level, comparator, follow-up duration, outcome measures, effect estimates where available, and principal findings. Information regarding potential unintended consequences of financial incentives was also recorded when reported. Discrepancies were resolved through discussion and consensus, with consultation with a third reviewer when necessary.

Risk of Bias Assessment

The risk of bias of the included RCTs and cluster RCTs was independently assessed by two reviewers using the Cochrane RoB 2 tool [7]. Assessment covered the five prespecified domains: bias arising from the randomisation process, bias due to deviations from intended interventions, bias due to missing outcome data, bias in measurement of the outcome, and bias in selection of the reported result. Because blinding of healthcare providers and participants is often difficult or impractical for payment and financial incentive interventions, lack of blinding was not considered an automatic indication of high risk of bias. Instead, the potential consequences of knowledge of intervention allocation were assessed within the relevant RoB 2 domains, taking into account the nature of the intervention and the outcome being measured. Overall judgements of low risk of bias, some concerns, or high risk of bias were based on the RoB 2 signalling questions and domain-level assessments. Any disagreements were resolved through discussion and consensus.

Certainty of Evidence

The certainty of evidence for the principal outcomes was assessed using the GRADE framework [8]. Evidence was initially considered according to study design and was assessed for potential downgrading because of risk of bias, inconsistency, indirectness, imprecision, and publication bias. Overall certainty was classified as high, moderate, low, or very low. Because the evidence base was heterogeneous and relatively small, publication bias was considered qualitatively where formal assessment was not appropriate. Summary GRADE assessments were prepared for the principal outcome domains.

Data Synthesis

The data synthesis approach was prespecified according to the characteristics of the available evidence. A quantitative meta-analysis was considered during synthesis planning; however, statistical pooling was not undertaken because of substantial clinical and methodological heterogeneity across the included studies. Heterogeneity was observed in the type and intensity of financial intervention; the level at which the intervention was delivered (provider, practice, facility, or patient); comparator conditions; healthcare settings; study populations; follow-up duration; outcome definitions; and methods of outcome measurement. The reported outcomes also varied considerably, including provider behaviour, preventive screening, vaccination, chronic disease management, healthcare utilisation, patient engagement, and other quality-of-care indicators. Because sufficiently comparable intervention-outcome combinations were not available to generate clinically interpretable pooled estimates, the findings were synthesised narratively. Studies were grouped according to the type and level of financial intervention and the principal outcome domain, and the direction, magnitude, consistency, and clinical relevance of reported effects were considered. Particular attention was given to differences between provider-directed, facility-level, and patient-directed financial interventions and to reported unintended consequences. The narrative synthesis was used to identify consistent patterns of benefit, areas of uncertainty, and important differences across healthcare settings.

Results

Study Selection

**Figure 1:**
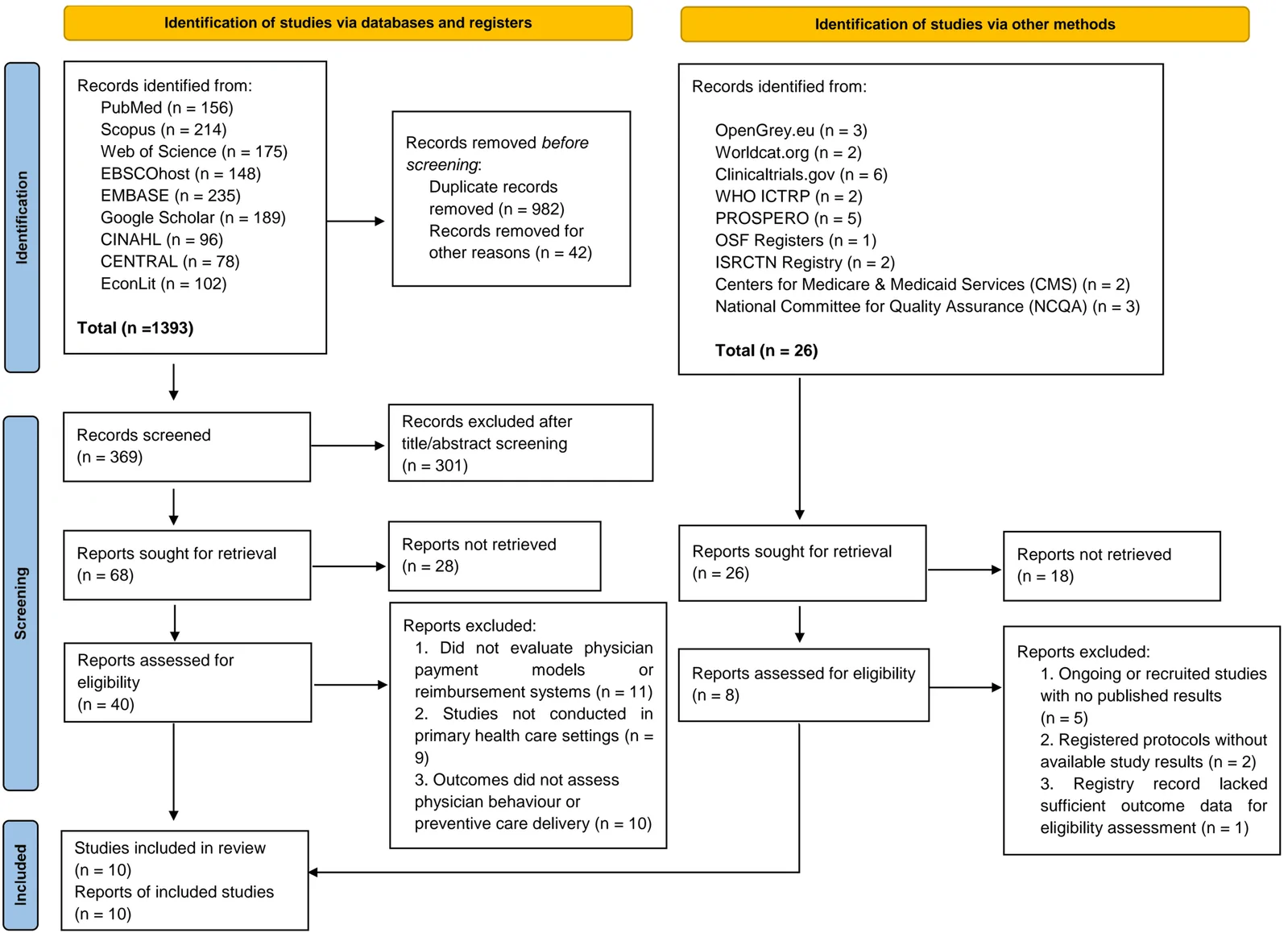
PRISMA 2020 flow diagram of study selection The PRISMA 2020 flow diagram illustrates identification, screening, eligibility assessment, reasons for exclusion, and final inclusion of studies in the qualitative synthesis. PROSPERO: Prospective Register of Systematic Reviews; ICTRP: International Clinical Trials Registry Platform; OSF: Open Science Framework; CINAHL: Cumulative Index to Nursing and Allied Health Literature; ISRCTN: International Standard Randomised Controlled Trial Number

The literature search identified 1,419 records from electronic databases and supplementary sources, including 1,393 records retrieved through database searches and 26 records identified from trial registries and other sources. Following the removal of 982 duplicate records and 42 records removed for other reasons, 369 records underwent title and abstract screening. Of these, 301 records were excluded because they did not satisfy the predefined eligibility criteria. Full-text reports were sought for 68 records, of which 28 could not be retrieved. The remaining 40 reports were assessed for eligibility, resulting in the exclusion of 30 reports for reasons including inappropriate study design, ineligible interventions, unsuitable outcomes, or failure to meet the inclusion criteria. In parallel, 26 reports identified through other sources were screened, of which 18 could not be retrieved, and eight were assessed for eligibility; all eight were excluded because they were ongoing or recruited studies without published results, registered protocols without available study results, or registry records lacking sufficient outcome data for eligibility assessment. Ultimately, 10 studies comprising both RCTs and cluster RCTs were included in the qualitative synthesis. The study selection process is illustrated in Figure 1.

Characteristics of the Included Studies

The review included 10 studies (RCTs and cluster RCTs) published between 2003 and 2023, representing a broad range of physician payment interventions implemented in primary healthcare settings. Eight studies were conducted in the United States, while one study each originated from Nigeria and Switzerland, reflecting evidence from both high-income and lower-resource healthcare systems.

The interventions evaluated included P4P, physician financial incentives, performance-based financing, direct facility financing, practice-level incentive programmes, and patient-directed financial incentive strategies. Study settings included community-based primary care practices, integrated physician networks, Veterans Affairs healthcare facilities, diabetes prevention programmes, safety-net clinics, and national primary healthcare systems. Sample sizes varied considerably, ranging from small provider-based trials to large studies involving more than 297,000 patients. The principal outcomes assessed across studies included physician adherence to clinical guidelines, preventive service delivery, quality-of-care indicators, chronic disease management, healthcare utilisation, preventive screening, immunisation, and patient engagement. A detailed summary of the characteristics and principal findings of the included studies is presented in Table 1.

**Table 1:** Characteristics and principal findings of the included studies This table summarises the characteristics of the included studies, the physician payment model evaluated, the primary outcome assessed, and the principal findings relevant to physician behaviour and preventive care delivery. RCT: Randomised controlled trial; P4P: Pay-for-performance

Study	Country	Study design	Payment model/intervention	Primary outcome	Principal finding
Roski et al., 2003 [14]	USA	Cluster RCT	Clinic-level financial incentives	Smoking cessation practices	Improved tobacco-use identification; limited effect on smoking cessation outcomes
Bardach et al., 2013 [15]	USA	Cluster RCT	P4P	Cardiovascular preventive care	Improved blood pressure control, antithrombotic prescribing, and smoking cessation interventions
Petersen et al., 2013 [16]	USA	Cluster RCT	Individual- and practice-level financial incentives	Hypertension management	Individual physician incentives improved guideline-concordant blood pressure management
Bradley et al., 2017 [17]	USA	RCT	Patient-directed cash incentives	Primary care attendance	Financial incentives increased attendance at initial primary care visits.
Navathe et al., 2019 [18]	USA	RCT + cohort	P4P with bonus modifications	Quality-of-care indicators	Larger bonus payments improved quality performance; behavioural framing offered no additional benefit
Desai et al., 2020 [19]	USA	Cluster RCT	Financial incentives	Diabetes prevention	Improved programme attendance but not overall weight loss
Green et al., 2020 [20]	USA	Pilot RCT	P4P	Preventive screening	Increased completion of incentivized preventive measures but raised concerns regarding documentation quality
Khanna et al., 2021 [21]	Nigeria	Cluster RCT	Performance-based financing	Maternal and child health services	Improved preventive service delivery compared with routine financing
Meier et al., 2021 [22]	Switzerland	Cluster RCT	Financial incentives	Diabetes quality indicators	Quality improvements persisted temporarily after incentive withdrawal
Schickedanz et al., 2023 [23]	USA	Pilot RCT	Financial coaching intervention	Preventive paediatric care	Reduced missed preventive visits and vaccination delays.

Effects of Physician Payment Models on Physician Behaviour and Preventive Care

Overall, the included studies suggest that physician payment reforms were associated with improvements in several aspects of preventive healthcare delivery. However, the magnitude of benefit differed according to the payment model, healthcare setting, and outcome evaluated.

P4P and Physician Incentive Programmes

Pay-for-performance was the most frequently investigated reimbursement strategy. Across most studies, financial incentives were associated with improved compliance with evidence-based clinical guidelines, increased delivery of preventive services, enhanced chronic disease monitoring, and better performance on quality-of-care indicators. Positive effects were observed for cardiovascular risk management, hypertension care, diabetes monitoring, preventive screening, and smoking cessation interventions. However, behavioural modifications such as loss-aversion framing and social comparison generally produced little additional improvement beyond conventional financial incentives. One pilot study also reported that gains in incentivised performance measures were accompanied by increased documentation and a reduction in some aspects of consultation quality, indicating the potential for unintended consequences.

Performance-Based Financing

Performance-based financing and direct facility financing were evaluated in a large cluster RCT conducted in Nigeria. Both financing approaches improved maternal and child health services when compared with routine funding mechanisms. Although performance-based financing demonstrated greater improvements in institutional deliveries, direct facility financing produced similar or better outcomes for several preventive indicators, including childhood immunisation coverage and utilisation of insecticide-treated bed nets. These findings suggest that simplified financing mechanisms may achieve meaningful improvements in preventive healthcare without the administrative complexity associated with performance-linked payment systems.

Patient-Directed Financial Incentives

Several studies examined patient-directed financial incentives as complementary strategies to improve preventive healthcare utilisation. Financial incentives increased attendance at initial primary care appointments, improved enrolment and participation in diabetes prevention programmes, and reduced missed preventive visits among children. Collectively, these findings indicate that patient-focused financial interventions may complement physician payment reforms by improving engagement with preventive healthcare services.

Overall Synthesis

Across the included trials, physician payment reforms were consistently associated with improvements in process-related measures of healthcare quality, including preventive screening, guideline adherence, vaccination delivery, smoking cessation counselling, hypertension management, and chronic disease monitoring. In contrast, evidence supporting improvements in patient-centred clinical outcomes was less consistent. Considerable variation in intervention characteristics, outcome definitions, healthcare settings, and duration of follow-up prevented quantitative pooling of results, making a narrative synthesis the most appropriate approach.

Methodological Quality and Risk of Bias

The methodological quality of the included studies was evaluated using the Cochrane RoB 2 tool [7]. Overall, six studies were judged to have some concerns, while four studies were classified as having a high risk of bias. No study fulfilled the criteria for an overall low risk of bias.

The principal methodological limitations included unavoidable lack of blinding of healthcare professionals, deviations from intended interventions, incomplete outcome data resulting from participant attrition, and potential bias associated with documentation-based outcome assessment. In contrast, selective reporting bias was generally low because most studies reported prespecified outcomes. The detailed methodological assessment is summarised in Table 2.

**Table 2:** Summary of methodological quality assessment using the Cochrane RoB 2 tool The five domains include the randomization process, deviations from intended interventions, missing outcome data, measurement of outcomes, and selection of reported results. Overall risk of bias was classified as low, some concerns, or high. RoB: Risk of bias

Study	Randomization	Intervention deviations	Missing outcome data	Outcome measurement	Selective reporting	Overall risk of bias
Roski et al., 2003 [14]	Some concerns	Some concerns	High	Some concerns	Low	High
Bardach et al., 2013 [15]	Some concerns	High	Some concerns	High	Low	High
Petersen et al., 2013 [16]	Low	Some concerns	Some concerns	Low	Low	Some concerns
Bradley et al., 2017 [17]	Some concerns	Some concerns	High	Low	Low	High
Navathe et al., 2019 [18]	Some concerns	Some concerns	Some concerns	Low	Low	Some concerns
Desai et al., 2020 [19]	Low	Some concerns	High	High	Low	High
Green et al., 2020 [20]	Low	Some concerns	Low	Low	Some concerns	Some concerns
Khanna et al., 2021 [21]	Low	Some concerns	Low	Some concerns	Low	Some concerns
Meier et al., 2021 [22]	Low	Some concerns	Some concerns	Some concerns	Low	Some concerns
Schickedanz et al., 2023 [23]	Low	Some concerns	Low	Low	Some concerns	Some concerns

Certainty of Evidence

The certainty of evidence ranged from very low to moderate, with no outcome supported by high-certainty evidence. Most outcomes were downgraded because of methodological limitations, particularly risk of bias and imprecision. Moderate-certainty evidence was available for selected outcomes, including cardiovascular preventive care, primary care attendance in the randomised incentive comparison, diabetes prevention programme attendance, and maternal and child preventive services in the performance-based financing versus direct facility financing comparison. Other outcomes were rated low or very low certainty because of risk of bias, imprecision, inconsistency, or indirectness. Overall, the available evidence suggests that payment and financial incentive interventions may improve selected preventive care processes; however, confidence in the magnitude and consistency of these effects remains limited. A summary of the certainty of evidence is presented in Table 3.

**Table 3:** Summary of certainty of evidence assessed using the GRADE framework GRADE: Grading of Recommendations Assessment, Development and Evaluation; P4P: Pay-for-performance

Outcome	Study	Comparison	Certainty	Main reason for downgrading
Smoking cessation practices and outcomes	Roski et al., 2003 [14]	Financial incentives vs. control	Low	Risk of bias; imprecision
Cardiovascular preventive care indicators	Bardach et al., 2013 [15]	P4P vs. control	Moderate	Risk of bias
Guideline-concordant hypertension management	Petersen et al., 2013 [16]	Physician incentive vs. control	Low	Risk of bias; inconsistency
Primary care attendance	Bradley et al., 2017 [17]	$25/$50 vs. $0 incentive	Moderate	—
Primary care attendance	Bradley et al., 2017 [17]	Incentive vs. non-incentivized group	Low	Risk of bias; imprecision
Quality-of-care performance	Navathe et al., 2019 [18]	Larger P4P bonus vs. comparator	Low	Risk of bias; imprecision
Diabetes prevention programme attendance	Desai et al., 2020 [19]	Financial incentive vs. attention control	Moderate	Risk of bias
Weight loss at 12 months	Desai et al., 2020 [19]	Financial incentive vs. attention control	Low	Risk of bias; imprecision
Completion of incentivized preventive measures	Green et al., 2020 [20]	P4P vs. flat-fee control	Very low	Risk of bias; indirectness; imprecision
Maternal and child preventive services	Khanna et al., 2021 [21]	Performance-based financing vs. direct facility financing	Moderate	Inconsistency
Maternal and child preventive services	Khanna et al., 2021 [21]	Financing interventions vs. routine financing	Low	Risk of bias; inconsistency
Diabetes quality indicators	Meier et al., 2021 [22]	Financial incentive vs. feedback-only	Low	Risk of bias; imprecision
Preventive visits and vaccination outcomes	Schickedanz et al., 2023 [23]	Financial coaching vs. usual care	Low	Risk of bias; imprecision

Discussion

This systematic review synthesised evidence from 10 studies that included both RCTs and cluster RCTs and evaluated payment and financial incentive interventions intended to influence healthcare delivery and preventive care in primary healthcare settings. The included interventions encompassed P4P, performance-based financing, direct facility financing, physician-level financial incentives, and patient-directed financial incentives. Overall, the evidence suggests that financial incentive and value-oriented payment strategies may improve selected processes of care, particularly physician adherence to evidence-based practices and delivery of preventive services. However, effects on patient-centred clinical outcomes were less consistent. These findings indicate that financial incentives may be more effective in modifying measurable healthcare processes than in producing short-term changes in downstream clinical outcomes. The findings are broadly consistent with previous systematic reviews reporting modest improvements in selected quality-of-care indicators following financial incentive interventions [4,5,15,16,18-22].

The most consistent effects across the included studies were observed for process-of-care outcomes, including guideline adherence, preventive screening, hypertension management, diabetes monitoring, smoking cessation counselling, and delivery of preventive services. Several trials, including those by Roski et al., Bardach et al., Petersen et al., Meier et al., and Khanna et al., reported improvements in specific preventive or quality-of-care indicators following implementation of financial incentive interventions [14-16,21,22]. These findings are consistent with previous reviews suggesting that financial incentives may influence healthcare processes more readily than long-term clinical outcomes or healthcare costs [4,5]. A possible explanation is that changes in clinician behaviour and healthcare delivery may occur relatively early, whereas measurable changes in morbidity, mortality, or healthcare utilisation may require longer periods of follow-up.

The comparatively limited and inconsistent effects on patient-centred outcomes should therefore be interpreted cautiously. Clinical outcomes are influenced by multiple factors beyond the payment mechanism, including patient adherence, socioeconomic circumstances, health literacy, healthcare accessibility, continuity of care, and organisational capacity. In addition, the follow-up periods of many included studies were relatively short, generally ranging from approximately six to 24 months, which may have limited the ability to detect longer-term changes in chronic disease control and other downstream outcomes [17-23]. Thus, the absence of consistent improvements in patient-centred outcomes should not necessarily be interpreted as evidence that financial incentives are ineffective, but rather as an indication that changes in provider behaviour and care processes may not immediately translate into measurable clinical benefits.

The effectiveness of financial incentives also appeared to vary according to intervention design and implementation. In particular, the RCT by Navathe et al. found greater improvement in quality performance with larger bonus payments, whereas behavioural incentive framing, including loss-aversion approaches, provided limited additional benefit [18]. However, this finding was derived from a specific intervention and should not be interpreted as evidence that larger financial incentives are universally more effective. Differences in incentive magnitude, target behaviour, payment structure, duration, baseline performance, and organisational context may all influence the response to financial incentives. Previous systematic reviews similarly suggest that the effects of P4P programmes are heterogeneous and depend substantially on programme design and implementation characteristics [4,5].

Evidence from low- and middle-income settings provides additional insight into alternative approaches to financing primary healthcare. The cluster RCT conducted in Nigeria reported improvements in maternal and child health service delivery with both performance-based financing and direct facility financing compared with routine financing [21]. The finding that direct facility financing produced comparable improvements in selected outcomes while involving a different administrative structure highlights the importance of considering contextual and organisational factors when designing financing interventions. However, because only a limited number of included studies were conducted in low- and middle-income settings, these findings should not be generalised broadly across diverse health systems. Further comparative research is required to determine whether particular financing approaches offer sustainable advantages in resource-constrained settings [1,24].

Several included studies also evaluated patient-directed financial incentives and reported improvements in outcomes such as attendance at primary care visits, participation in diabetes prevention programmes, and uptake of preventive services [17,19,23]. These findings are important because they demonstrate that financial incentives can operate through more than one pathway. Interventions directed at patients may complement provider-level incentives by addressing demand-side barriers to preventive care. Nevertheless, these interventions differ conceptually from physician remuneration and should be distinguished from provider payment models when interpreting the evidence. Their inclusion also broadens the interpretation of the present review from physician remuneration alone to financial incentive strategies operating at different levels of primary healthcare delivery.

Financial incentives may also produce unintended consequences. The pilot RCT by Green et al. reported increased completion of incentivised preventive measures but also raised concerns regarding increased documentation and changes in aspects of interpersonal communication [20]. Similar concerns have been identified in previous literature, including the possibility that providers may preferentially focus on incentivised activities or devote greater attention to documentation requirements [4]. These potential unintended effects emphasise the importance of selecting performance indicators that encourage comprehensive, patient-centred care rather than narrowly rewarding isolated activities.

The methodological quality of the included evidence also warrants careful consideration. In the RoB 2 assessment, no study was judged to have an overall low risk of bias; six studies were classified as having some concerns and four as having a high risk of bias. Importantly, the absence of blinding was not automatically interpreted as evidence of high risk of bias. Risk judgements were made within the relevant RoB 2 domains and considered the likely impact of deviations from intended interventions, missing outcome data, outcome measurement, and selection of reported results. Concerns were particularly relevant where knowledge of intervention allocation could plausibly influence implementation, outcome assessment, or participant behaviour. Consequently, the overall certainty of evidence should be interpreted with caution, particularly for outcomes supported by studies with important methodological limitations.

The GRADE assessment further indicated that certainty of evidence across the principal outcome domains ranged from very low to moderate. Downgrading was primarily related to risk of bias, inconsistency, imprecision, and, where applicable, indirectness. The variability in certainty reinforces the need to distinguish between the direction of observed effects and confidence in the magnitude and reliability of those effects. Although several studies demonstrated favourable effects on process-of-care outcomes, the available evidence does not provide sufficiently high-certainty evidence to conclude that financial incentive interventions consistently improve long-term patient-centred outcomes.

A meta-analysis was not performed because the included studies differed substantially in the characteristics of the interventions, comparators, healthcare settings, target populations, outcome definitions, follow-up periods, and reported measures of effect. The interventions also operated at different levels, including physician, facility, and patient levels. Although statistical pooling can sometimes be undertaken despite clinical and methodological heterogeneity, pooling estimates that represent materially different interventions or outcome constructs could produce a summary effect that lacks meaningful clinical interpretability. Therefore, the review used a structured narrative synthesis, grouping studies according to the type and level of financial intervention and examining patterns of effects across physician behaviour, preventive care processes, healthcare utilisation, and patient-centred outcomes. This approach was considered more appropriate for the heterogeneous evidence base than generating an overall pooled estimate.

From a policy perspective, the findings suggest that financial incentives may contribute to improvements in selected aspects of primary healthcare quality, but they should not be viewed as a standalone solution for improving healthcare delivery. The potential benefits of payment and incentive interventions are likely to depend on programme design, organisational infrastructure, baseline performance, administrative requirements, and the broader healthcare context. Integration with audit and feedback, electronic health records, multidisciplinary quality-improvement programmes, and patient-engagement strategies may enhance their effectiveness [1,24]. Importantly, policymakers should also consider potential unintended consequences, administrative burden, equity implications, and the possibility of preferential attention to incentivised activities when designing such programmes.

By focusing on RCTs and cluster RCTs, this review sought to strengthen causal inference compared with evidence derived predominantly from observational studies. Nevertheless, the evidence base remains limited and heterogeneous. Future research should prioritise adequately powered multicentre RCTs with longer follow-up periods, standardised outcome definitions, transparent reporting of intervention components and implementation characteristics, and economic evaluations. Greater representation of low- and middle-income countries is also needed to determine whether the effects observed in predominantly high-income healthcare systems are transferable to different financing and primary-care contexts. Future studies should additionally distinguish clearly between physician-level payment interventions, facility-level financing, and patient-directed incentives to allow more precise assessment of their respective effects.

Limitations

Several limitations should be considered when interpreting the findings. First, only 10 eligible studies featuring both RCTs or cluster RCTs were included, resulting in a relatively small evidence base. Second, substantial clinical and methodological heterogeneity was present across payment and financial incentive interventions, healthcare settings, target populations, outcome definitions, and follow-up periods. This heterogeneity limited the clinical interpretability of quantitative pooling; therefore, a structured narrative synthesis was undertaken rather than a meta-analysis. Third, the included interventions were not restricted to physician remuneration alone and included physician-level, facility-level, and patient-directed financial incentives. Consequently, the findings should be interpreted as evidence regarding broader financial incentive and payment interventions in primary healthcare rather than as definitive evidence for physician remuneration models alone. Fourth, most included studies were conducted in the United States, which may limit generalisability to healthcare systems with substantially different financing structures, workforce characteristics, and primary-care organisation. Fifth, no included study was judged to have an overall low risk of bias, with several studies raising concerns related to the randomisation process, deviations from intended interventions, missing outcome data, outcome measurement, or selection of reported results. Finally, the certainty of evidence ranged from very low to moderate, and variation in intervention design and implementation limited the ability to identify a single payment or incentive strategy that can be considered consistently superior across different primary healthcare settings.

## Conclusions

This systematic review indicates that payment and financial incentive interventions in primary healthcare may improve selected processes of care, particularly preventive service delivery, adherence to evidence-based practices, and chronic disease management. However, the evidence for improvements in patient-centred clinical outcomes was less consistent, and the overall certainty of evidence ranged from very low to moderate because of risk of bias, inconsistency, imprecision, and heterogeneity across interventions and outcomes. The findings suggest that financial incentives may contribute to improvements in primary healthcare quality but should not be considered a standalone strategy; their effectiveness is likely to depend on intervention design, implementation context, and integration with broader quality-improvement and patient-engagement approaches. Further well-designed, adequately powered multicentre RCTs with longer follow-up, standardised outcome measures, and economic evaluations are needed to determine the long-term effectiveness, cost-effectiveness, and generalisability of different payment and financial incentive strategies across diverse healthcare systems.

## Appendices

Appendix A

Table 4 features a summary of the complete database-specific search strategies, search limits, and search dates. 

**Table 4:** Summary of the electronic database and grey literature search strategy MeSH: Medical Subject Headings; P4P: Pay-for-performance; FFS: Fee-for-service, PROSPERO: Prospective Register of Systematic Reviews; ICTRP: International Clinical Trials Registry Platform; OSF: Open Science Framework; CINAHL: Cumulative Index to Nursing and Allied Health Literature; ISRCTN: International Standard Randomised Controlled Trial Number; TITLE-ABS-KEY: Title, abstract, and keywords

Information source	Search strategy summary	Limits/filters applied
PubMed/MEDLINE	Combined Medical Subject Headings (MeSH) and free-text terms related to physician/provider payment models, reimbursement, financial incentives, P4P, value-based payment, FFS, capitation, physician/provider behaviour, clinical practice, preventive care, and primary healthcare, using Boolean operators (AND/OR)	Humans; English; January 2000-December 2025
Scopus	TITLE-ABS-KEY search combining terms related to payment models, provider/physician payment, financial incentives, value-based payment, P4P, reimbursement, physician/provider behaviour, clinical practice, primary care, and preventive care using Boolean operators	English; Articles, Reviews, Conference Papers; 2000-2025
Web of Science	Topic search (TS) combining terms related to payment models, provider payment, reimbursement, value-based payment, P4P, physician/provider behaviour, practice patterns, primary care, and preventive services	English; 2000-2025
EBSCOhost	Title/Abstract search using terms related to payment models, reimbursement, provider payment, financial incentives, P4P, value-based payment, physician/provider behaviour, primary care, and preventive care	English; 2000-2025
Cochrane CENTRAL	Search combining terms related to payment models, provider payment, reimbursement, P4P, value-based payment, FFS, capitation, primary healthcare, and preventive care	English; 2000-2025
CINAHL	Combination of CINAHL subject headings (MH) and free-text terms related to primary healthcare, preventive health services, physicians, payment models, provider payment, financial incentives, P4P, value-based payment, FFS, capitation, and salary	English; 2000-2025
Embase	Combination of Emtree controlled vocabulary and free-text terms related to physician reimbursement, FFS, capitation, P4P, value-based payment, physicians/clinical practice, primary healthcare, and preventive health services.	English; 2000-2025
Google Scholar	Multiple simplified searches combining payment models, physician payment, value-based payment, P4P, physician/provider behaviour, primary care, and preventive care. The first 200-300 records retrieved by relevance were screened.	English; 2000-2025
EconLit	Search combining terms related to payment models, reimbursement, provider payment, financial incentives, value-based payment, P4P, physicians/providers, primary healthcare, and preventive care	English; 2000-2025
ClinicalTrials.gov	Searches using combinations of payment model, provider payment, P4P, value-based payment, capitation, and primary healthcare	English; January 2000-December 2025
WHO ICTRP	Searches using combinations of payment model, physician reimbursement, P4P, value-based payment, and primary care	English; January 2000-December 2025
OpenGrey	Searches using combinations of payment model, physician reimbursement, value-based payment, P4P, primary care, and preventive care	English; January 2000-December 2025
WorldCat	Searches using combinations of payment model, physician payment, value-based payment, primary care, and preventive care	English; January 2000-December 2025
PROSPERO	Separate searches using payment model, physician payment, value-based payment, P4P, primary care, and preventive care to identify relevant systematic review protocols and completed reviews	English; January 2000-December 2025
OSF Registries	Searches using combinations of payment model, physician reimbursement, primary care, and preventive care	English; January 2000-December 2025
ISRCTN Registry	Searches using combinations of payment model, P4P, primary care, and value-based payment	English; January 2000-December 2025
Reference-list and citation screening	Reference lists of eligible studies and relevant systematic reviews were manually screened, with citation tracking performed where appropriate to identify additional potentially eligible studies.	No additional database limits
